# Poly(vinylalcohol) (PVA) Assisted Sol-Gel Fabrication of Porous Carbon Network-Na_3_V_2_(PO_4_)_3_ (NVP) Composites Cathode for Enhanced Kinetics in Sodium Ion Batteries

**DOI:** 10.3390/polym14010149

**Published:** 2021-12-31

**Authors:** Junghoon Yang, Duyoung Choi, Kwang-Seok Kim, Dae Up Kim, Jungpil Kim

**Affiliations:** Carbon & Light Materials Application Research Group, Korea Institute of Industrial Technology, Jeonju 54853, Korea; duychoi@kitech.re.kr (D.C.); ore21@kitech.re.kr (K.-S.K.); dukim@kitech.re.kr (D.U.K.)

**Keywords:** Na_3_V_2_(PO_4_)_3_, porous carbon network, composite material, cathode material, sodium ion batteries

## Abstract

Na_3_V_2_(PO_4_)_3_ is regarded as one of the promising cathode materials for next-generation sodium ion batteries, but its undesirable electrochemical performances due to inherently low electrical conductivity have limited its direct use for applications. Motivated by the limit, this study employed a porous carbon network to obtain a porous carbon network–Na_3_V_2_(PO_4_)_3_ composite by using poly(vinylalcohol) assised sol-gel method. Compared with the typical carbon-coating approach, the formation of a porous carbon network ensured short ion diffusion distances, percolating electrolytes by distributing nanosized Na_3_V_2_(PO_4_)_3_ particles in the porous carbon network and suppressing the particle aggregation. As a result, the porous carbon network–Na_3_V_2_(PO_4_)_3_ composite exhibited improved electrochemical performances, i.e., a higher specific discharge capacity (~110 mAh g^−1^ at 0.1 C), outstanding kinetic properties (~68 mAh g^−1^ at 50 C), and stable cyclic stability (capacity retention of 99% over 100 cycles at 1 C).

## 1. Introduction

Rapid advancements in renewable energy generation, including solar, wind, and geothermal systems, have encouraged the improvements in large-scale energy storage systems (ESS) for their efficient utilization [1,2,3,4]. Lithium ion batteries (LIBs) have been considered as one of the most reliable ESS due to their high energy density. However, the growing concern about the limited reserve of lithium resources and an increase in price restricts the use of LIBs in the large-scale ESS, because they need inexpensive and efficient systems to have reliability and sustainability. Rechargeable sodium ion batteries (SIBs) have recently been regarded as the most promising candidates as next-generation rechargeable battery systems by virtue of the earth-abundant distribution of sodium resources [5,6,7]. However, the electrochemical properties of SIBs have exhibited several drawbacks that are mainly originated from sluggish ion mobility and severe structural degradation during the insertion/de-insertion of sodium ions because of the larger ionic radius of sodium ions compared with that of lithium ions [8]. Therefore, it is a top priority to find suitable host materials for SIBs with reliable electrochemical properties. Among various cathode materials including layer-structured transition metal oxide (Na_x_TMO_2_) [9,10,11,12,13,14] and transition metal hexacyanoferrate [15,16,17], poly-anion type phosphate-based materials such as sodium vanadium phosphate (Na_3_V_2_(PO_4_)_3_, NVP) have attracted attention due to their reliable electrochemical properties [18,19,20].

In general, alkali vanadium phosphate materials have two different polymorphs including the monoclinic phase and the rhombohedral phase. The structural difference between these two phases is originated from the interconnection ways between VO_6_ octahedra and PO_4_ tetrahedra. In the case of Li_3_V_2_(PO_4_)_3_, a monoclinic phase is preferred, whereas NVP has a rhombohedral structure, indicating the ionic size of alkali ions is the most important factor for deciding a thermodynamically stable phase [21,22,23]. The rhombohedral NVP belongs to the Na superionic conductor (NASICON) family, and it has an open-structured framework that allows the fast diffusion of sodium ions (high power capability) through a large interstitial lattice. The strong inductive effect of PO_4_^3−^ anion lifts the energy level of the V^3+^/V^4+^ redox couple that results in a relatively high operating voltage (~3.4 V vs. Na/Na^+^, hereafter) of the material. Furthermore, NVP provides stable cyclic stability based on robust structural stability during electrochemical processes. However, the intrinsically low electronic conductivity of NVP due to the strong covalent bond in the phosphate group generally results in poor electrochemical performances, when the materials are used without an electrical conductor coating, which has become the main limitation for practical applications [24,25]. The most common strategy for improving the electronic conductivity is to form a carbon-coating layer [26]. For instance, Jian et al. reported the improved electrochemical properties of NVP by forming a carbon-coating layer on the surface of the particles [27]. Generally, the carbon layer can provide pathways for electrons, and thus, it increases electronic conductivity when it is covered the surface of NVP. However, we should carefully consider the ion diffusivity of the carbon-coating layer, because it is not a good ion conductor. As the thickness of the carbon-coating layer became thicker, the sodium ion diffusivity of the composite might be decreased because it should pass-through the thick surface carbon-coating layer before it reaches NVP crystal [28,29,30]. Therefore, the double sidedness of the carbon-coating strategy for electronic conductivity and ion diffusivity must be considered in the formation of carbon–NVP composites to have maximized benefits and the corresponding electrochemical properties.

Here, we proposed a straightforward strategy for the development of an NVP and carbon composite that exhibited a porous structure by taking advantage of chelating interactions between NVP precursors and poly(vinylalcohol) (PVA). The advantages of the porous structure are related to the formation of an interconnected carbon framework and forming isolated NVP particles embedded in the porous carbon matrix. In detail, the Porous NVP/C composite had potential electrochemical advantages: (i) the thin and homogeneous carbon coating layer on NVP particles enhanced the interfacial electrical conductivity and sodium ion transfer; (ii) the suppression of NVP particle aggregation facilitated the uniform response of the particles; and (iii) the porous carbon framework provided a larger surface area that increased the active redox surface of the composites. Consequently, the Porous NVP/C composite exhibited outstanding electrochemical properties as cathode material for SIBs. In this study, in order to study the improvement of electrochemical properties, the characteristics of the carbon layer such as the porosity, specific surface area, and the coating layer thickness were investigated with the conventional simple carbon-coating method.

## 2. Materials and Methods

### 2.1. Materials Preparation

For the preparation of materials, NH_4_VO_3_ (Sigma-Aldrich, St. Louis, MO, USA), NH_4_H_2_PO_4_ (Sigma-Aldrich, St. Louis, MO, USA), and CH_3_COONa (Sigma-Aldrich, St. Louis, MO, USA) were mixed with a 14 wt % citric acids (Sigma-Aldrich, St. Louis, MO, USA) solution and stirred for 2 h at 70 °C. PVA-2000 (Sigma-Aldrich, St. Louis, MO, USA) was used as a porous carbon network source to prepare Porous NVP/C composites. As a reference material, NVP/C composites were synthesized without PVA-2000. The above solution was further heated to 100 °C to get homogeneous powder. The obtained precursor powders were heated at 800 °C for 6 h in an Ar atmosphere with a ramp rate of 5 °C/min.

### 2.2. Material Characterization

The morphologies of the materials were analyzed using a field-effect scanning electron microscope (FE-SEM; JEOL JSM-6700F, Tokyo, Japan) and a high-resolution transmission electron microscope (HR-TEM; JEOL JEM-3010, Tokyo, Japan). Powder X-ray diffraction (XRD; Rigaku Ultima IV, Tokyo, Japan) analysis was carried out to identify the structural property. The carbon content in the composites was determined by thermogravimetric analysis (STA6000, Perkin Elmer, Waltham, MA, USA). A Raman spectroscope (MonoRa 750i/ELT10000; the spectral excitation was produced by using the 488 nm line of an argon-ion laser, and the incident light was focused on the surface of the structure using a 50× microscope objective) was used to characterize carbon and NVP. The surface area and pore characteristics of the materials were calculated by the Brunauer–Emmett–Teller (BET) analysis and the Barrett–Joyner–Halenda (BJH) method, respectively (BELSORP-mini II, MicrotracBEL, Osaka, Japan). The elemental information was determined by X-ray photoelectron spectroscopy (XPS; PHI 5000 VersaProbe, ULVAC-PHI, Kanagawa, Japan). The compositional information of inorganic components in the Porous NVP/C composites was analyzed by an inductively coupled plasma-atomic emission spectrometer (ICP-AES; OPTIMA 8300, Perkin-Elmer, Waltham, MA, USA).

### 2.3. Electrochemical Analysis

The electrode was prepared by mixing the active materials (80 wt %), acetylene black (10 wt %, TIMCAL, Switzerland), and polyvinylidene fluoride (PVdF, 10 wt %) in an N-methylpyrrolidone (NMP, Sigma-Aldrich, St. Louis, MO, USA) solvent. The slurry was coated onto an Al foil using a doctor blade and then dried in a vacuum oven at 120 °C for 5 h. After drying, the electrodes were pressed and then punched into a rectangle shape. The average loading density of the active material on the current collector was ~2.5 mg cm^−^^2^. The electrochemical properties of the prepared electrodes were evaluated using CR 2032 coin-type cells that were assembled in an Ar-filled glovebox. A Na metal foil was used as a counter electrode, and 1 M NaPF_6_ in ethylene carbonate (EC) and diethyl carbonate (DEC) (1:1, *v*/*v*) was employed as an electrolyte. The electrochemical performances of the assembled coin-type cells were tested by galvanostatic charge–discharge analysis at room temperature in the voltage range of 2.7–4.0 V (Na/Na^+^) (WBCS 3000; Wonatech, Republic of Korea). The galvanostatic intermittent titration technique (GITT) measurement was performed at an electrochemical workstation by applying a constant current flux of 0.05 C for 20 min, followed by an open-circuit equilibration time of 2 h.

## 3. Results

**Figure 1:**
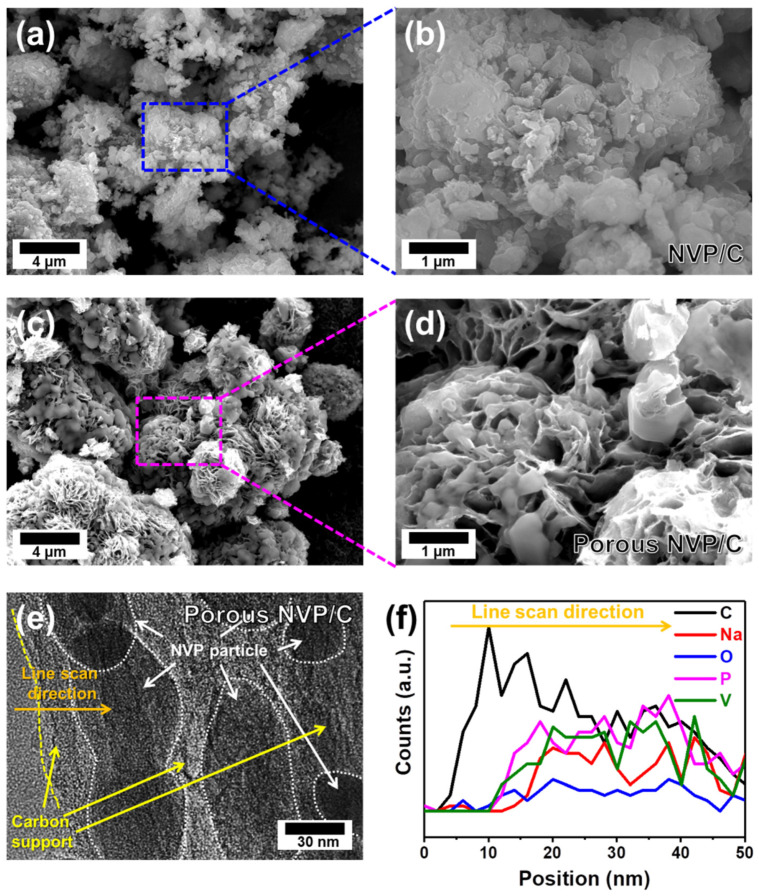
SEM images of NVP/C composites (**a**,**b**) and Porous NVP/C composites (**c**,**d**). (**e**) TEM image of Porous NVP/C composites. (**f**) Elemental line scans for C, Na, O, P, and V along the orange-colored line in (**e**).

NVP/C and Porous NVP/C composites were prepared by the sol-gel synthesis method. Initially, NH_4_VO_3_ was added to a solution of citric acid, and the solution color changed gradually from yellow to dark blue. The color change was due to the strong oxidizing ability of VO_3_^−^ ions. In the solution, VO_3_^−^ (V^5+^) ions were reduced to VO^2+^ (V^3+^), while C_6_H_8_O_7_ molecules oxidized to C_5_H_6_O_5_ molecules and carbonyl groups in the C_5_H_6_O_5_ could chelate VO^2+^ ions. Then, NH_4_H_2_PO_4_ and CH_3_COONa were added under constant stirring. As the VO^2+^ ions and the chelators had stable interactions, the chelated VO^2+^ ions could act as sites for the nuclei formation of NVP precursors. In order to form a porous carbon network for Porous NVP/C composites, PVA was added to the solution before high-temperature heat treatment, while NVP/C composites were prepared without PVA [31,32]. It is known that the hydroxyl groups can interact with metal ions and the random arrangement of the polymer chain enhance the mixing of metal ions when PVA is added. The prepared solution was heat-treated to crystallize the NVP structure and make the surrounding carbon-coating layers. In the case of the Porous NVP/C composites, the carbon precursor (both citric acid and PVA) formed a surface carbon-coating layer on the surface of the NVP particles and the cross-links network between neighboring particles, whereas the NVP/C composites using only citric acid as the chelating agent had a carbon-coating layer without porous networks in the composites. The effect of adding PVA on the morphological characteristics was analyzed by SEM analysis, as presented in Figure 1. In the case of the NVP/C composites in Figure 1a,b, the uneven particle sizes with a range from the sub-micron to micron scale of the NVP particles were shown. The particle aggregation and the uneven carbon coating layer were clearly observed in the NVP/C composites with no formation of the porous carbon-support network between particles. In the case of the Porous NVP/C composites, however, it clearly showed the formation of a porous carbon network that looked like a flower shape as a result of adding PVA in the precursor solution (Figure 1c). Compared with the NVP/C composites, the porous carbon networks effectively restricted the aggregation of NVP particles by dispersing the particles in a continuous carbon network matrix (Figure 1d). The TEM image in Figure 1e provided a more detailed picture of the structure of the Porous NVP/C composites, which can be clearly identified by the differences in brightness. In the composites, relatively lighter parts represented conductive carbon networks, and the relatively darker part showed crystalline NVP particles. Notably, small NVP particles (30–100 nm in diameter) were enveloped by carbon networks, which would be beneficial for fast electron conduction. Furthermore, the elemental line scan result of the Porous NVP/C composites along the orange-colored arrow in Figure 1e provided further information for distinguishing elements between the carbon network and NVP particles. As shown in Figure 1f, following the orange arrow from left to right, only the carbon signals appeared strongly from the beginning of the line scan from 0 nm to about 10 nm. The elemental signals for Na, O, V, and P started to appear around 10 nm of the line scan analysis, and it was also confirmed that the carbon signal was relatively weak as the line scan progressed. A relatively weaker carbon signal near 30 nm indicated that the carbon layer was thin in the region where NVP particles were present.

**Figure 2:**
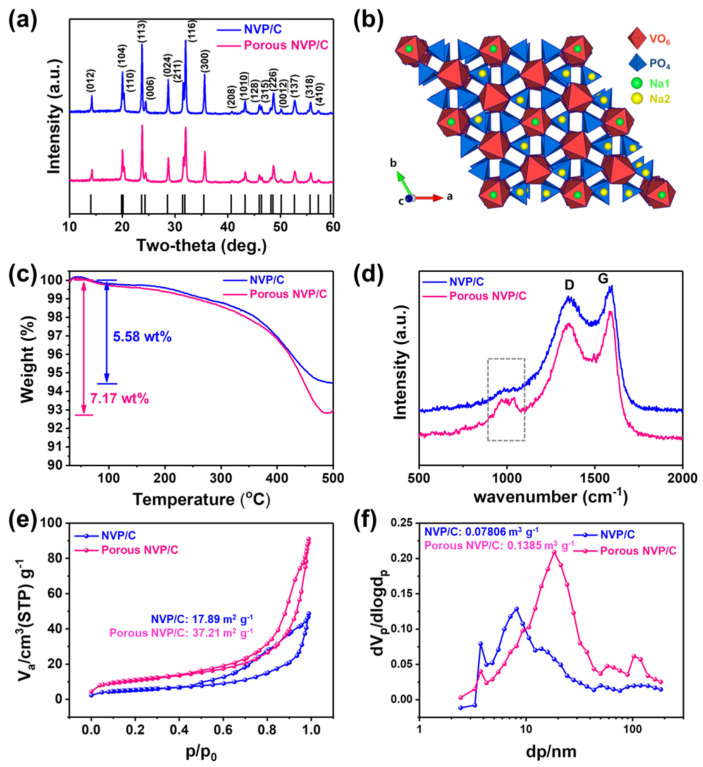
(**a**) XRD patterns of the NVP/C composites and the Porous NVP/C composites. (**b**) Crystal structure of NVP. (**c**) TGA results of the NVP/C and Porous NVP/C composites for analyzing the carbon contents in the composites. (**d**) Raman spectra of the NVP/C and Porous NVP/C composites. (**e**) N_2_ adsorption–desorption isotherm. (**f**) Pore size distribution of the NVP/C and Porous NVP/C composites.

Figure 2a shows the XRD patterns of the NVP/C and Porous NVP/C materials. All of the observed diffraction peaks can be well-matched with the rhombohedral structure of Na_3_V_2_(PO_4_)_3_ with the R-*3c* space group. The crystalline structure of Na_3_V_2_(PO_4_)_3_ is illustrated in Figure 2b. It is composed of octahedral VO_6_ and tetrahedral PO_4_, which formed a 3D [V_2_(PO_4_)_3_]^3−^ framework. Sodium ions are occupied in two different crystallographic sites with different oxygen environments: M1 and M2 interstitial sites of six- and eight-fold coordination, respectively. In the previous report, the sodium ions at the M2 site have a relatively weaker connection to surrounding oxygen atoms compared with that of sodium ions at the M1 site. Therefore, the sodium ions located at the M2 site are easy to be extracted from the structure, and thus, they are involved in the electrochemical behavior [33]. NVP has a V^3+^/V^4+^ redox reaction corresponding to the insertion and de-insertion of two sodium ions, since sodium ions, which are more stable than the sodium ion at the M1 position, are known to be immobile in electrochemical processes. More information for the characteristics of the carbon layer was further analyzed. The carbon contents of both composites were measured by the TGA method, as shown in Figure 2c. Generally, the initial weight loss at ~500 °C was originated from the conversion of carbon species into CO_2_ and the corresponding mass loss of carbon, when it was analyzed in the air atmosphere. The analyzed carbon contents were 5.58 and 7.17 wt % for the NVP/C and Porous NVP/C composites, respectively. The larger amount of the carbon content for the Porous NVP/C composites is reasonable, because they contained more carbon precursors (PVA) than the NVP/C composites in the precursor solution as we mentioned in the experimental section. In other words, the concentrations of NVP in the composites were 94.42 and 92.83 wt % for the NVP/C and Porous NVP/C composites, respectively. The Raman analysis was conducted to further determine the nature of carbon and NVP crystal in the composites. As shown in Figure 2d, two strong carbon signals located at ~1345 and 1587 cm^−^^1^ demonstrated the coexistence of amorphous carbon and graphitic carbon in the composites [34,35,36,37,38]. The characteristic Raman signals of NVP crystals were also represented by bands from 100 and 1100 cm^−^^1^. The bands located at ~960 and 1040 cm^−1^ were related to the stretching vibrations of PO_4_^3−^ tetrahedra [39]. Interestingly, as highlighted by the grey box, the Raman signal related to PO_4_^3−^ was only observed in the Porous NVP/C composites. Considering the limits in the depth of laser of the Raman spectrometer that was mainly restricted to the surface region of the sample due to its low energy, the signal of the Raman spectra of the composites should be affected by the thickness of the carbon layer. Therefore, the Raman spectra of the NVP/C composites, which showed only the signal of carbonaceous species, demonstrated a thicker carbon layer formed on the NVP particles. That is, it can be seen that the Porous NVP/C composites exhibited Raman signals for PO_4_^3^^−^, because it had thin carbon layers sufficient for the Raman laser to penetrate. Although the TGA results showed that Porous NVP/C composites had a higher carbon content, the presence of a thinner carbon layer indicated that more efficient distribution of the carbon network in the composites by adding PVA. The efficient distribution here means the formation of a thin flower-like porous carbon network as we observed from electron microscopic analysis. The BET and BJH methods were also conducted to determine the specific surface area, the total pore volume, and the pore size distribution. The specific surface areas of the NVP/C and Porous NVP/C composites were measured to be 17.89 and 37.21 m^2^ g^−^^1^, respectively. In addition, the total pore volume of the Porous NVP/C composites was 0.1385 m^3^ g^−^^1^, which was about two times higher than that of the NVP/C composites (0.07806 m^3^ g^−^^1^). The relatively larger specific surface and pore volumes should be favorable for the electrolyte to penetrate and beneficial to have a higher interface between NVP particles and electrolytes.

**Figure 3:**
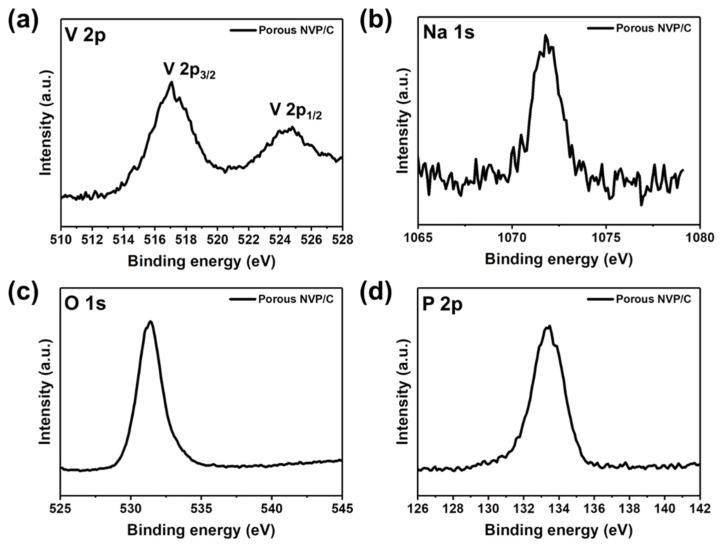
XPS spectra of the Porous NVP/C composites: (**a**) V 2p; (**b**) Na 1s; (**c**) O 1s; and (**d**) P 2p.

The surface information regarding the valence states of elements of the Porous NVP/C composites was evaluated by XPS analysis, as shown in Figure 3. The V 2p signal of the Porous NVP/C composites appeared at the binding energies of ~517 and 524 eV, corresponding to V 2p_3/2_ and V 2p_1/2_, respectively. The peak positions of the V 2p signals were well-matched with previously reported values for Na_3_V_2_(PO_4_)_3_ or Li_3_V_2_(PO_4_)_3_, demonstrating the trivalent state of vanadium (V^3+^) in the Porous NVP/C composites as expected from the theoretical composition of the Na_3_V_2_(PO_4_)_3_ material [22,40]. The Na 1s signal located at ~1071 eV was a typical signal for sodium ions in the vanadium (V^3+^) phosphate structure. In addition, the O 1s signal at 531.2 eV and the P 2p signal at ~133.4 eV were attributed to the oxygen and phosphorous atoms of the (PO_4_)^3−^ phosphate group [41]. Considering that the O 1s peak of the oxide-based material is generally located at ~528.5 eV, the higher binding energy of O 1s peak for the Porous NVP/C composites can be understood as more covalent characteristics of P-O bonds [42,43]. The P 2p spectra with no other peaks at ~129 eV also showed that the phosphorus was present as the phosphate (PO_4_)^3−^ group rather than the phosphide group. On the basis of the above XPS results, the Porous NVP/C composites were successfully synthesized as we intended to. To further confirm the chemical composition of the Porous NVP/C composites, the ICP-AES analysis was conducted. The converted atomic % of Na and V were 0.857 and 0.573, respectively, and thus, the calculated chemical composition was assumed to be Na_2.99_V_2_(PO_4_)_3_ that was quite similar to the expected theoretical composition.

**Figure 4:**
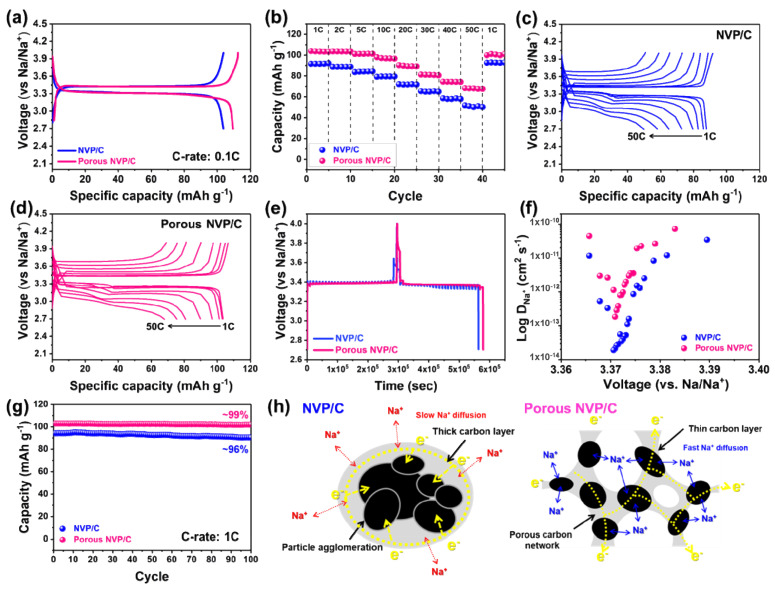
Electrochemical performances validation of the NVP/C and Porous NVP/C composites. (**a**) Galvanostatic charge–discharge curves measured at 0.1 C in a voltage window of 2.7–4.2V. (**b**) Rate capability measured from 1 to 50 C. (**c**) Voltage profile of the NVP/C composites at a specific capacity range of 0–120 mAh g^−1^. (**d**) Voltage profile of the Porous NVP/C composites at a specific capacity range of 0–120 mAh g^−1^. (**e**) Galvanostatic intermittent titration technique (GITT) curves measured using the current flux at 0.05 C for 20 min for the relaxation time of 2 h. (**f**) Sodium ion diffusion coefficient during the discharge process of the NVP/C and Porous NVP/C composites calculated from the GITT results. (**g**) Cyclic performance at a rate of 1 C over 100 cycles. (**h**) Schematic illustration comparison between the NVP/C and Porous NVP/C composites for illustrating the beneficial sodium ion and electron transfer in the Porous composite structure toward the improved electrochemical properties.

The electrochemical properties of the two samples (NVP/C and Porous NVP/C composites) as cathode materials for NIBs were carried out in a voltage window of 2.7–4.2 V. It is worth noting that the redox reaction of both samples in the galvanostatic charge and discharge process was essentially related to the two-phase reaction between Na_3_V_2_(PO_4_)_3_ and NaV_2_(PO_4_)_3_, as indicated by the well-formed plateau at ~3.4 V [18,24]. As mentioned earlier, the two sodium ions extraction and insertion from and into the structure are related to sodium ions that are occupying the M2 site, while the residual one sodium ion at the M1 site remained immobilized in the structure during electrochemical reactions. Therefore, the theoretical capacity of NVP materials was assumed to be ~117.8 mAh g^−^^1^ according to the V^3+^/V^4+^ redox reaction. As shown in Figure 4a, the measured discharge capacities of the NVP/C and Porous NVP/C composites at 0.1 C (1 C = 118 mA g^−^^1^) were ~103 and ~110 mAh g^−^^1^, respectively. Interestingly, the discharge capacity of the Porous NVP/C composites was very close to the theoretical capacity. Considering that the TGA analysis results showed that the Porous NVP/C composite had a slightly lower NVP content, the higher capacity of the Porous NVP/C composite might be seem strange, because only NVP participated in the electrochemical reaction in the applied voltage window condition. As the carbonaceous species were not involved in electrochemical behaviors in the applied voltage window, the mass of carbon in the composite did not contribute to the capacity but rather was taken into account when calculating the specific capacity, thus easy to result in a decrease in the capacity. Therefore, the higher reversible capacity of the Porous NVP/C composite can be understood as a result of effective electron conduction and sodium ion transfer. As mentioned earlier, the carbon layer on the NVP particles was essential to improve electrical conductivity of the composites, but it also acted as an additional layer that hindered the movement of sodium ions.

Therefore, it seemed that the reason for the Porous NVP/C composite exhibited a higher reversible capacity seems to be because it can form a uniform and thin carbon layer than that of the NVP/C composite. To further verify the merit of the porous composite structure, the rate capability of the NVP/C and Porous NVP/C composites were evaluated as shown in Figure 4b. Interestingly, the Porous NVP/C composite showed specific discharge capacities of ~103, 103, 101, 97, 90, 81, 74, and 68 mAh g^−^^1^ at C-rates of 1, 2, 5, 10, 20, 30, 40, and 50 C, respectively. The 68 mAh g^−^^1^ of capacity at 50 C was as high as ~61.8% of the discharge capacity measured at 0.1 C. In case of the NVP/C composite, it exhibited discharge capacities of ~91, 88, 83, 79, 71, 65, 57, and 50 mAh g^−^^1^ at C-rates of 1, 2, 5, 10, 20, 30, 40, and 50 C, respectively. It was clearly observed that the Porous NVP/C composite showed higher discharge capacities under all of the C-rate conditions than those of the NVP/C composite. Considering that the C-rate of 50 C was an extremely fast charging and discharging condition for the material to be charged and discharged within 72 s, the favorable environment of the Porous NVP/C composite for fast and efficient sodium ion diffusion and electron transfer was well demonstrated by its outstanding rate capability. The superior rate capability of the Porous NVP/C composite was also comparable to those in already reported works (Table 1) [44,45,46,47,48]. The clear differences in the voltage fade and the observed capacity with the increasing C-rate condition were directly compared by the galvanostatic charge and discharge profiles obtained at various C-rate conditions, as shown in Figure 4c,d. The improved rate capability of the Porous NVP/C composite was further investigated by the GITT technique (Figure 4e). The diffusion coefficient for sodium ions during the discharge process was calculated from the GITT curves according to Fick’s second law of diffusion as follows:

(1) $$\tilde{D}=\frac{4}{\pi \tau }{\left(\frac{m_{B}V_{M}}{M_{B}A}\right)}^{2}{\left(\frac{\Delta E_{s}}{\Delta E_{t}}\right)}^{2}\;\;\;\;\;\;\;(\tau \;\ll \;\frac{L^{2}}{\tilde{D}}),$$

where $\tilde{D}$ is the sodium ion diffusion coefficient, m_B_ is the mass of the active material, M_B_ is the atomic weight, V_M_ is the molar volume, A is the surface area of the electrode, $\Delta E_{t}$ is the total change of the voltage during the discharge at a constant current, and $\Delta E_{s}$ is the voltage difference between the equilibrium states before and after the current pulse. During the discharge process, as shown in Figure 4f, both the NVP/C and Porous NVP/C composites showed a “V”-shape of diffusion coefficient values that demonstrated the two-phase mechanism during the electrochemical process [49]. The calculated diffusion coefficient values of the Porous NVP/C composite (3.8 × 10^−13^–7.23 × 10^−11^) were higher than those of the NVP/C composite (3.5 × 10^−14^–3.4 × 10^−11^), and it demonstrated the merits of the porous carbon network for the improved rate capability. Figure 4g shows the cyclic performances of both the NVP/C and Porous NVP/C composites at a rate of 1 C over 100 cycles. After the cyclic test, NVP/C and Porous NVP/C maintain ~90.6 and 102.1 mAh g^−1^ of the discharge capacity that corresponded to 96% and 99% of the initial capacity, respectively. The superior cyclic stability of both the NVP/C and Porous NPV/C composites should be originated from the robust structural properties of the 3D crystal structure. The schematic illustration for the benefit of the porous composite structure is shown in Figure 4h. It should be attributed to the shorter ion transport pathway due to the formation of small-size particles that were evenly interconnected by a thin carbon-coating layer. The shorter diffusion length may facilitate the reaction kinetics by reducing the diffusion time according to the following equation of τ = L^2^/D, where τ is the diffusion time, L is the diffusion length, and D is the diffusion coefficient. Furthermore, the porous structure of the carbon network provided a larger contact area for electrolytes and composite materials based on its higher specific surface area and larger pore volume.

## 4. Conclusions

We have successfully fabricated a composite, in which NVP particles were homogeneously dispersed in a porous carbon matrix by adopting a PVA-assisted sol-gel fabrication method. The Porous NVP/C composite contained NVP particles (~30–100 nm of particle size in diameter) that were uniformly embedded in a porous and thin carbon network. The porous carbon network played multiple roles in the composite: it acted as barrier to prevent particle aggregation, provided sufficient electron conduction pathways that enabled overcoming the inherently low electronic conductivity of NVP and also provided a larger surface area for contacting an electrolyte solution. The merits of the porous structure formation were well demonstrated by its outstanding electrochemical properties of the Porous NVP/C composite when compared with that of the NVP/C composite. The Porous NVP/C composite exhibited a superior rate capability (discharge capacity of 68 mAh g^−1^ at 50 C) and also maintained ~99% of its initial discharge capacity at 1 C even after 100 cycles. Therefore, the polymer-assisted sol-gel processes for phosphate cathode materials can provide a method for fabricating porous carbon networks and cathode particles composites that promote efficient electron conduction and ion transfer.

## Figures and Tables

**Table 1:** Comparison of the rate capabilities of NVP cathodes between this work and other reports.

Composite	Preparation Method	Rate Capability	References
NVP/C	PVA-assisted sol-gel method	50 mAh g^−^^1^ at 50 C	This work
Porous NVP/C	PVA-assisted sol-gel method	68 mAh g^−^^1^ at 50 C	This work
Na_3_V_2_(PO_4_)_3_/C	Electrospinning	30 mAh g^−^^1^ at 30 C	[44]
NVP@C@HC	Sol-gel method	61 mAh g^−^^1^ at 50 C	[45]
Na_3_V_2_(PO_4_)_3_	Solid-state reaction method	71.9 mAh g^−^^1^ at 10 C	[46]
Na_3_V_2_(PO_4_)_3_/C	Soft-template method	54.3 mAh g^−^^1^ at 30 C	[47]
NVP/MCNTs	Sol-gel method	70 mAh g^−^^1^ at 10 C	[48]
